# Demoralization in the wake of the COVID-19 pandemic: Whereto the future for young Australians?

**DOI:** 10.1177/1473325020973332

**Published:** 2021-03

**Authors:** Patricia Fronek, Lynne Briggs

**Affiliations:** School of Human Services and Social Work, Griffith University, Southport, Australia; Law Futures Research Centre, Griffith University, Nathan, Australia; School of Human Services and Social Work, Griffith University, Southport, Australia

**Keywords:** COVID-19, young people, demoralization, unemployment, critical reflection

## Abstract

COVID-19 is changing lives. Less attention has been paid to the future of young people by governments since the onset of the pandemic. We suggest that young people are vulnerable to demoralization, a state of hopelessness and helplessness, during and post-COVID-19. This reflection drawn from journaling and ongoing reflexive conversation from December 2019 to April 2020 offers a critical perspective on the circumstances of young Australians that encompasses consideration of the structural factors that impact on health, life chances, equality and social justice as well as the personal aspects of reflection.

*This pandemic is not a war…It is a test of our humanity*

German President, Frank-Walter Steinmeier

COVID-19 is indeed testing how humanity is sustained while laying bare rampant individualism and community solidarity. Demoralization is an important concept in this health emergency as it relates to the loss of hope, helplessness and an inability to see a viable future. It affects many vulnerable people, yet it is a relatively rare concept in social work practice. Demoralization is too often confused with depression leading to misguided interventions which may not address core issues associated with the loss of hope and helplessness. One major difference is that people who are depressed cannot motivate themselves to action whereas demoralized people can be motivated only to be restricted by external factors such as structural barriers in society, illness or other insurmountable problems (Briggs and Fronek, 2019).

One needs only consider the writings of Viktor Frankl about hope in Nazi concentration camps or the years of detention endured by refugees to understand the concept of demoralization (Frankl, 2004). Demoralization which is not a mental illness does pose risks to mental health and, if unaddressed, can lead to mental health problems such as depression and in some cases, suicide (Briggs and Fronek, 2019). With the onset of the COVID-19 pandemic, there has been ample media coverage about maintaining mental health while in lockdown. People who have a home, are healthy and employed, are experiencing disruptions to normal life. For others, the present and future was far less certain. The focus of this reflection is on one group, young adults in Australia, made vulnerable to demoralization through unemployment, a circumstance worsened when intersected with pre-existing disadvantage or discrimination. Drawing on the work of Jan Fook, journaling and sharing reflections throughout the COVID-19 emergency from December 2019 to April 2020, we engaged in a critically reflexive process which encompassed structural factors impacting on young adults, challenged our assumptions as well as considering our own reactions to COVID-19 and the political responses to it (Fook and Gardner, 2007).

The Australian federal government initially chose a mitigation approach with an almost exclusive focus on the economy from the outset (Ferguson et al., 2020). Mitigation aims to maintain the economy, slow not eradicate disease, and to prevent overload on unprepared and underfunded health systems. However, this approach inevitably costs lives and allows some spread of the disease. Health-oriented strategies to ‘flatten the curve’ such as restricting large gatherings and imposing lockdown were only implemented two months after the first confirmed case (Evershed et al., 2020). In contrast, suppression strategies adopted early in countries such as Iceland, China and New Zealand, aimed to stop the spread of infection and save lives (Ferguson et al., 2020). Measures such as widespread testing, lockdown and quarantine, were described daily as ‘draconian’ by many Australian politicians and media.

By the 14th April, the number of cases and deaths were low compared to other countries (6,400 confirmed cases and 61 deaths) (Australian Government, 2020). Restrictive testing meant a true gauge of prevalence was difficult to determine. Eight hundred “excess deaths” in the first quarter suggests these figures were indeed underestimated (Bennett, 2020). Health messages were contradictory and confusing, delivered in a slow, trickle-down dribble while properly equipping hospitals and health workers was left to the States to eventually assume leadership over these issues.

In reality, no government should be surprised by this pandemic as such an emergency has long been predicted (Doherty, 2013; Morse et al., 2012). COVID-19 shone a spotlight on unprepared health systems. Governments in the United States, Australia, and the United Kingdom have focused on either resisting universal health coverage or seeking to dismantle it. Prior to April, we were all encouraged to maintain normal social participation including large sporting events favoured by the Prime Minister. Tourists were urged to keep coming, and contagion risks were downplayed. Political conservatism squarely placed responsibility for public health in the hands of businesses to choose their own responses and on individuals to manage themselves regardless of capacity, resources and power imbalances. There was no early national co-ordination to ensure basics such as how supermarkets would ensure fair food distribution and manage hoarding fixations on products such as toilet paper which is actually manufactured in Australia.

For those people and their families who were in good health and able to work from home, the challenges were very much first-world problems where movements were restricted, holidays cancelled, and families forced to spend time together. With exceptions such as those already isolated, homeless, mentally ill, caregiving, living with family violence, or at risk of child abuse or other social problems, stressors were increased but hardly represented hardship comparatively. Because the federal government remained hopeful that strict measures could be avoided, planning for predicted impacts such as unemployment were belated. It was not until mid-March that the government promised new time-limited income support with restrictive eligibility criteria to be implemented at the end of April. Generous packages for employers to keep people ‘employed’ among other business focused benefits were made available. Meanwhile redundancies continued. There is no argument that the economy is important and the situation complex, however social work values support prioritising lives. A study of past pandemics suggests that economic recovery is faster when lives are prioritized (Correia et al., 2020).

Before the pandemic, women and young people in Australia made up much of the underemployed, casual and part-time workforce and the rate of unemployment in younger people was double that of others in the population (Atkins et al., 2020; Coates et al, 2020; Dimov et al., 2020). Prior to COVID-19, 13.6 million young people aged under 24 years were engaged in various combinations of work and study (ABS, 2019). The Australian government predicted 1.4 million unemployed in a population of only twenty-six million as a result of the pandemic, which proved to be an underestimate (ABS, 2020).

Young adults have been hit hard (Atkins et al., 2020; Dimov et al., 2020). Many live from pay cheque to pay cheque with little or no savings to fall back on during a crisis. Job losses were significant which affected their ability to pay rent placing many in powerless positions with landlords and estate agents, risking homelessness and couch surfing. Not all young adults can live with their families and some tertiary students were already homeless or supporting dependents (Heerde et al., 2020). Students face considerable debt as governments have systematically shifted the cost of higher education from government to students and in the midst of the pandemic has again shifted costs to students, announced plans to double the cost of social work degrees, and reducing university funding.

Young people in insecure employment have been the first recessionary casualties. The longer a young person is unemployed, the more precarious future employability becomes, locking young people into a cycle of poverty and loss of career opportunities as recovery post-pandemic is estimated to take decades (Dimov et al., 2020). These factors, magnified for young people with pre-existing and intersecting disadvantage, impact negatively on self-perceptions, life opportunities, and physical and mental health (Dimov et al., 2020). It is well documented that stress, poverty and inequality affects transmission, susceptibility, severity and duration of viral illnesses (Janes et al., 2012). Although young adults are at less risk of COVID-19 complications, they can become seriously ill or die, and debilitating, chronic disabling conditions may result. Poor health, unemployment and a loss of hope for future job prospects are known factors that can lead to demoralization (Briggs and Fronek, 2019).

The lack of recognition of power imbalances and pre-existing inequalities are staggering. Rather than addressing the inadequacy of income support and including the already unemployed in new measures, a generous benefit was devised for the ‘deserving’, unemployed due to COVID-19, while the pre-COVID unemployed, the ‘undeserving’, continued to live below the poverty line (Fronek, 2017). A chasm was created between those people who had been employed for twelve months with an expectation of remaining in their jobs and those in more insecure work, international students, seasonal workers, those employed for less than twelve months and the already unemployed, perceived as not having ‘worked hard enough’.

Prior to COVID-19, homelessness, underemployment and financial distress were problems which became exponentially and rapidly exacerbated. We posit that many young people in the midst of this pandemic and for many years afterwards are at high risk of demoralization if they cannot achieve life goals, see a future for themselves or align their lives with hopes and dreams when facing long term unemployment or homelessness. Demoralization should therefore be considered when working with young adults in this uncertain climate while remaining aware of the potential confusion between demoralization and mental illness. Young adults may find it difficult to see a future that is equal or better than their parents and may feel helpless in the face of external barriers. Although many young Australians may be in better circumstances compared to many young adults in other countries, feeling fortunate or deprived is relative to those around us, and this perception deepens for those young people already disadvantaged.

Whereto the future? The emphasis should be on addressing practical concerns, supporting hope and building on strengths rather than working from a deficit perspective and implied individual dysfunction regardless of which theoretical perspective informs interventions. It is important to remain cognizant and to neither assume that young adults will cope nor inevitably fall into mental illness, and to recognize demoralization, loss of hope and helplessness, when present. Practical assistance that supports a path to reach unique, individual goals and developing life meaning support hope (Briggs and Fronek, 2019). Interventions for those who are struggling necessitates understanding how hopeful they are, the barriers they face, how to enhance purpose and meaning, and to believe in their strengths while meeting practical needs. Social workers are also tasked with addressing the macro as well as micro strategies to build hope for a future and pathways to that future.

Social workers are personally affected by COVID-19 which is challenging if not exhausting, while working within systems to help others, responding to crises, and often risking their own health in the course of their work. Addressing inequality and social injustice is difficult at a time when there may be little energy to do so. COVID-19 has taught us that social workers do need additional skills for health emergencies and education related to the social determinants of health, public health and pandemic preparedness (Janes et al., 2012).

As for our influences on this reflection, we have been angry and despairing at the prioritization of the economy over lives reflected in political decisions, and sceptical about the façade of political neutrality, and hopeful about others. This prioritization and delays in taking action were of particular concern to us. Despite success in ‘flattening the curve’ by April, the virus had spread in vulnerable communities, people have died unnecessarily, and the second wave was threatening. We also recognise that there are many vulnerable groups at risk of demoralization, not just young people, each with unique circumstances and equally affected by macro factors. The federal response had improved by April, and in our opinion, forced into being by several forces – a rapid increase in infections and deaths, celebrities and politicians (and relatives) becoming infected, insistent and concerned medical and public health voices, and State leadership taking action where voids existed albeit adding to public confusion through different messages. Although it is our expectation that COVID-19 will bring about many permanent changes across fields altering how we live and work, it is likely that politicking will return falling back on old ideologies in attempts to regain the pre-pandemic order. While realising the impact on people will not be over even with a vaccine or effective treatments, social workers must continue to question assumptions and reflect critically in order to best support our clients while learning from this pandemic experience as it will not be the last pandemic we face.

Unlike the German President, the Australian government calls this pandemic a war. In past wars, populations especially vulnerable populations, did not wear the fiscal brunt when war was over, rather governments focused on recovery. In post-war U.K. and Australia, governments built welfare states to meet health, education, employment and livelihood needs. People, especially young people, should not have to bear the costs which perpetuate ongoing struggles. Concern about humanity must over-ride the resurrection of failed systems.
